# The MNK1/2-eIF4E Axis as a Potential Therapeutic Target in Melanoma

**DOI:** 10.3390/ijms21114055

**Published:** 2020-06-05

**Authors:** Sathyen A. Prabhu, Omar Moussa, Wilson H. Miller, Sonia V. del Rincón

**Affiliations:** 1 Division of Experimental Medicine, McGill University, 1001 Decarie Boulevard, Montreal, QC H4A 3J1, Canada; sathyen.prabhu@mail.mcgill.ca (S.A.P.); omar.moussa@mail.mcgill.ca (O.M.); wilson.miller@mcgill.ca (W.H.M.J.); 2 Lady Davis Institute, Jewish General Hospital, McGill University, 3755 Côte Ste-Catherine Road, Montreal, QC H3T 1E2, Canada; 3 Department of Oncology, McGill University, 845 Sherbrooke St W, Montreal, QC H3A 0G4, Canada; 4 McGill Centre for Translational Research in Cancer (MCTRC), McGill University, 3755 Côte Ste-Catherine Road, Montreal, QC H3T 1E2, Canada; 5 Rossy Cancer Network, McGill University, 1980 Sherbrooke Ouest, #1101, Montreal, QC H3H 1E8, Canada

**Keywords:** melanoma, MNK1, eIF4E, translation, immunotherapy

## Abstract

Melanoma is a type of skin cancer that originates in the pigment-producing cells of the body known as melanocytes. Most genetic aberrations in melanoma result in hyperactivation of the mitogen activated protein kinase (MAPK) and phosphoinositide 3-kinase (PI3K) pathways. We and others have shown that a specific protein synthesis pathway known as the MNK1/2-eIF4E axis is often dysregulated in cancer. The MNK1/2-eIF4E axis is a point of convergence for these signaling pathways that are commonly constitutively activated in melanoma. In this review we consider the functional implications of aberrant mRNA translation in melanoma and other malignancies. Moreover, we discuss the consequences of inhibiting the MNK1/2-eIF4E axis on the tumor and tumor-associated cells, and we provide important avenues for the utilization of this treatment modality in combination with other targeted and immune-based therapies. The past decade has seen the increased development of selective inhibitors to block the action of the MNK1/2-eIF4E pathway, which are predicted to be an effective therapy regardless of the melanoma subtype (e.g., cutaneous, acral, and mucosal).

## 1. Classification of Melanoma Molecular Subtypes

Melanoma is the deadliest of all skin cancers and has its origins in the pigment-producing cells of the body known as melanocytes. Specific mutations in melanoma may be more predominant within particular pathological subtypes. For instance, The Cancer Genome Atlas (TCGA) defined three genomic subtypes of cutaneous melanoma based on the mutation status of BRAF, NRAS, NF1, and a fourth subgroup termed triple wild-type [1]. Acral and mucosal melanomas, on the other hand, harbor distinct mutations, namely in the gene encoding the c-KIT receptor tyrosine kinase (RTK) [2], while uveal melanomas often present with mutations in GNAQ/11 [3].

## 2. Frequently Occurring Mutations in Melanoma and Molecular Significance

**Figure 1:**
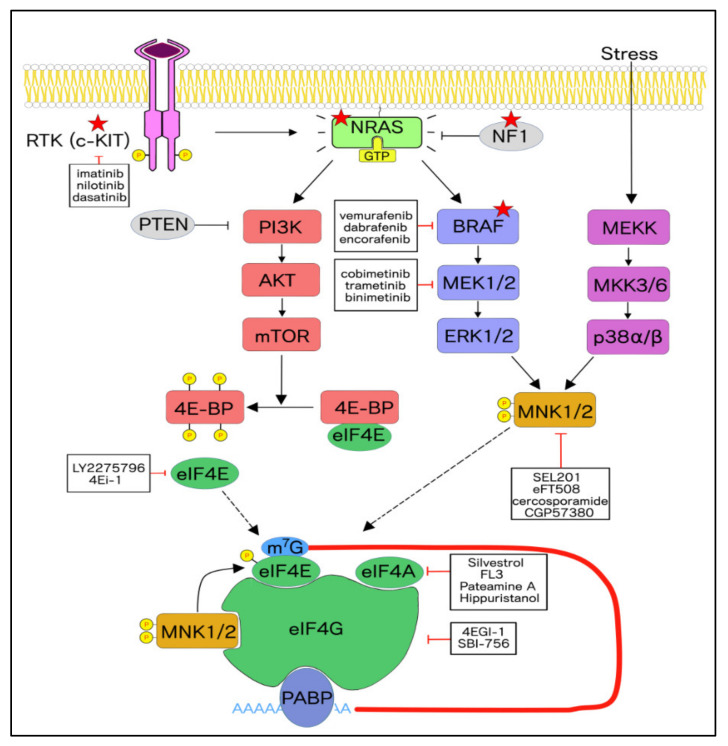
Schematic of the MNK1/2-eIF4E axis. The red stars on the NRAS, RAF, and NF1 indicate the three common subtypes of cutaneous melanoma. Mutations in BRAF result in the hyperactivation of the mitogen-activated protein kinase (MAPK) pathway while mutations in NRAS or NF1 result in the hyperactivation of both MAPK and phosphoinositide 3-kinase (PI3K) pathways. Activating mutations in c-KIT results in hyperactivation of Ras and downstream effector pathways. Signaling down the MAPK pathway results in the activation of MNK1/2 and signaling down the PI3K/Akt pathway results in the hyperphosphorylation of 4E-BP and the resultant release of eIF4E to associate with eIF4G. MNK1/2 bind to eIF4G and phosphorylate eIF4E at Ser209. This results in enhanced translation of certain mRNAs. MNK1 and MNK2 may also be phosphorylated by p38 through stress-mediated signals.

BRAF mutations are mostly caused by single nucleotide substitutions, of which approximately 90% occur at codon 600, where a valine residue is swapped with glutamic acid (BRAF^V600E^) [4]. This specific mutation results in the constitutive activation of the BRAF oncoprotein and sustained activation of the MEK/ERK pathway (Figure 1), while also conferring insensitivity to negative feedback regulation of the mitogen-activated protein kinase (MAPK) pathway [5]. BRAF mutations account for approximately 52% of cutaneous melanoma mutations, compared to only 6% in mucosal melanoma [6]. While BRAF mutations are the most prevalent in cutaneous melanoma, NRAS mutations display more aggressive phenotypes with increased mitotic rate, thicker primary tumors, and poorer prognosis [7,8]. Mutations in NRAS account for approximately 28% of cutaneous melanoma, of which more than 80% harbor a glutamine-to-leucine substitution (NRAS^Q61L^), thus compromising the intrinsic GTPase activity of NRAS and increasing its GTP-bound form [6]. GTP-bound Ras proteins activate the downstream effectors RAF and phosphoinositide 3-kinase (PI3K) (Figure 1). The abundance of Ras in its GTP-bound form also depends on the presence of the tumor suppressor NF1, a Ras GTPase-activating protein (GAP) (Figure 1). Mutations in the tumor suppressor NF1 are observed in approximately 14% of cutaneous melanoma, of which 63% are characterized by a loss-of-function [6]. Tumors that are negative for BRAF, NRAS, and NF1 mutations are termed triple wild-type and include a wide array of altered genes that may play a role in tumorigenesis. For instance, mutations in KIT, the gene encoding the c-KIT receptor, account for 22% of triple wild-type tumors. Oncogenic alterations in KIT include translocations within exons 11 and 13 (L576P and K624E, respectively), thus resulting in constitutive activation of c-KIT and subsequent activation of the PI3K/AKT and MAPK pathways [9] (Figure 1).

## 3. Targeted Therapies for Specific Molecular Subtypes in Melanoma

Several therapies have been designed to target the most frequent mutations in cutaneous melanoma. Drugs inhibiting the kinase activity of BRAF^V600E^ include vemurafenib, dabrafenib, and encorafenib. Vemurafenib was the first Food and Drug Administration (FDA)-approved BRAF^V600E^ inhibitor following a phase-III randomized clinical trial that was conducted in 675 patients [10]. In that study, treatment of advanced melanoma patients with vemurafenib improved overall survival and progression-free survival when compared to dacarbazine, a chemotherapeutic agent that was the first-line therapy approved for metastatic melanoma since 1972 [11]. While monotherapy using BRAF inhibitors appeared to be an exciting alternative to chemotherapy, responses were often temporary with resistance developing at a median of approximately seven months [12]. Resistance mechanisms to BRAF inhibition include reactivation of the MAPK pathway and activation of the PI3K/Akt pathway in approximately 70% and 22% of advanced tumors, respectively [13]. Furthermore, BRAF inhibition can paradoxically activate wild-type CRAF [14], and continued monotherapy with vemurafenib following the onset of resistance may further support tumor progression [15,16]. Thus, numerous combination therapies with MEK inhibitors were proposed to prevent or delay the onset of resistance to BRAF inhibition [17,18,19]. A number of resistance mechanisms to the combination therapy of BRAF and MEK inhibition have been characterized (reviewed in [20]). Some of these include BRAF amplification, oncogenic *NRAS* mutations, and *MEK1*/*2* mutations [20], in addition to a PAX3-mediated upregulation of MITF in approximately 80% of melanoma during early stages of resistance [21]. Moreover, NF1 loss of function may also confer resistance to MEK and BRAF inhibition [22]. Loss of other tumor suppressors such as *PTEN*, *TP53,* or *CDKN2A* may also account for the increased aggressiveness of *BRAF*-mutated tumors. For instance, co-occurrence of *BRAF^V600E^* and loss of *PTEN* manifest in approximately 20% of melanomas and show increased metastatic potential [23]. While several options are available for the treatment of BRAF-driven melanoma, limited targeted therapies are available for *NRAS*-mutated tumors, given the difficulties in directly targeting the Ras GTPase [24]. While inhibiting farnesylation of Ras may prevent its translocation to the plasma membrane and forestall activation of downstream effectors, farnesyltransferase inhibitors (FTIs) have shown no clinical benefit in advanced melanoma [25]. On the other hand, binimetinib, a MEK1/2 inhibitor, has shown improved progression-free survival when compared to dacarbazine in patients with advanced melanoma who harbor *NRAS*-mutations [26]. Conversely, resistance to MEK inhibition in *NRAS*-mutant melanoma may be mediated through activation of the anti-apoptotic cAMP/MITF/Bcl-2 pathway [27]. Other inhibitors of the MAPK pathway, including pan-RAF and ERK inhibitors, have been shown to decrease tumorigenesis in *NRAS*-mutated tumors [27,28,29]. In *c-KIT*-mutant melanoma, several RTK inhibitors have been tested in the clinical space, with comparable overall response rates (ORR) reported for imatinib (23.3%) and nilotinib (26.2%), and slightly lower rates for dasatinib (18.2%) [30,31,32]. In a phase-II clinical trial conducted in 28 patients with melanoma harboring *c-KIT* mutations or amplifications, treatment with imatinib yielded a durable response rate of 16%, with responses lasting more than one year [33]. More recently, ponatinib has been shown to exhibit greater potency than imatinib in inhibiting tumor growth in melanomas harboring *KIT* mutations, likely because of an increased ponatinib-KIT affinity [34]. Whereas targeted therapy against c-KIT has been effective in treating gastrointestinal stromal tumors (GIST) [35], its inhibitory activity is far less impressive in c-KIT-mutant melanoma, and responses tend to be short lived, with a median time to progression of three months [33]. Once again, mechanisms of resistance hamper the therapeutic benefits of RTK inhibitors, including amplification or overexpression of *KIT* [36], other simultaneous activating alterations in NRAS [36], and secondary mutations in the activation loop of c-KIT [37]. Furthermore, the L576P mutation in *KIT,* represented in approximately 34% of *KIT* mutations, confers poor sensitivity to imatinib in GIST [38]. In the context of melanoma where the L576P is the most common *KIT* mutation, patients show increased sensitivity to dasatinib [39]. Melanoma cells expressing dual activating mutations in *KIT* (e.g., L576P/T670I or A829P) while being resistant to imatinib, nilotinib, and dasatinib, did exhibit increased sensitivity to dual inhibition of the MAPK and PI3K pathways [37].

### 3.1. Rationale for Targeting the MNK1/2-eIF4E Axis in Cancer

Current targeted therapies in melanoma generally exhibit limited clinical efficacy, given the ability of tumors to develop resistance mechanisms [20]. One way that cancer cells adopt resistance is by hijacking the function of downstream effector proteins, sometimes involving the activation of parallel signaling pathways [40]. For instance, a convergence point downstream of the MAPK and the PI3K/AKT/mTOR pathways, arguably two of the most important signaling pathways in melanoma, is the eukaryotic initiation factor 4F (eIF4F) complex, which regulates mRNA translation initiation (Figure 1). Components of the eIF4F complex include (1) eIF4A, a DEAD-box RNA-helicase responsible for unwinding mRNA secondary structures, (2) eIF4E, which binds the 7’methylguanosine cap (m^7^G) at the 5’ end of mRNAs, and (3) eIF4G, a scaffold protein that interacts with eIF4E and eIF4A. The PI3K-AKT/mTOR pathway signals directly to eIF4E via the phosphorylation of eIF4E-binding proteins (4E-BPs). Hypophosphorylated 4E-BPs sequester eIF4E from binding to eIF4G, thus preventing formation of the translation initiation complex, while phosphorylation of 4E-BPs by mTOR releases eIF4E and activates translation [41] (Figure 1). Translation of specific subsets of mRNAs, including those encoding oncogenes, is further activated via the phosphorylation of eIF4E by mitogen-activated protein kinase (MAPK)-interacting kinases 1 and 2 (MNK1/2), downstream of MAPK activation [42]. MNK1/2 are the only kinases responsible for phosphorylating eIF4E on Ser209 [43,44].

Increased levels of eIF4E are associated with poor prognosis in many cancer types including breast [45], melanoma [46], prostate [47], gallbladder [48], colorectal adenocarcinoma [49], and hepatocellular carcinoma [50] and correlate with advancing tumor grade in squamous cell carcinoma [51] and esophageal cancer [52]. Moreover, the phosphorylation of eIF4E is tightly regulated and plays an important role in cell proliferation and metastasis [53,54]. Increased phospho-eIF4E levels is an independent prognostic factor in astrocytomas [55], NSCLC [56], and nasopharyngeal carcinoma [57], while also being associated with disease progression in melanoma [58] and prostate cancer [59]. Increased levels of phospho-eIF4E were also observed in gastric and colorectal cancers [60], whereas overexpression of MNK1 in epithelial ovarian cancer correlates with phospho-eIF4E levels and poor clinical outcome [61]. Our research has shown that *KIT*-mutant melanoma patients have increased levels of MNK1 and phospho-eIF4E, and in breast cancer increased levels of p-MNK1 were associated with high-grade ductal carcinoma in situ [62,63]. Moreover, while upstream components of the MAPK and PI3K pathways often show a heterogeneous expression pattern in tumors, the expression of phospho-eIF4E and phospho-4E-BP are more diffuse within the tumor and are overexpressed in breast cancer [64]. While eIF4E is required for cap-dependent mRNA translation, a genetically engineered eIF4E haploinsufficient mouse prevented cellular transformation but maintained normal development [65]. MNK1/2 and phospho-eIF4E are also dispensable for normal murine development [42,59,62]. Thus, whereas using BRAF and MEK inhibitors in metastatic melanoma patients caused severe adverse events (reviewed in [66]), pharmacologically inhibiting MNK1/2 kinases represents a promising therapeutic option, as limited side-effects and toxicities are expected upon abrogation of MNK1/2 activity. Together, these previously published works support the MNK1/2-eIF4E axis and eIF4F complex as promising therapeutic targets in cancer.

### 3.2. Structural and Functional Differences Between MNK1/2 Isoforms

**Figure 2:**
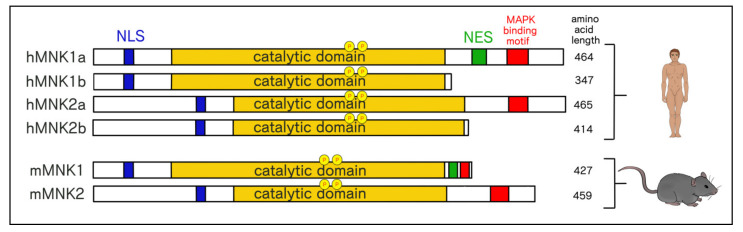
Schematic representation of the core features of human and murine mitogen activated protein kinase-interacting kinases 1 and 2 (MNK1 and MNK2). In humans, MNK1 and MNK2 are spliced into ”a” and “b” isoforms whereas in mice MNK1 and MNK2 are not spliced. NLS, nuclear localization signal; NES, nuclear export signal.

MNK1 and MNK2 are serine-threonine kinases encoded by two genes, *MKNK1* and *MKNK2,* respectively. In humans, each *MKNK* may be alternatively spliced into ”a” and ”b” isoforms, while in mice only the full length, or “a”, isoform has been reported [67,68] (Figure 2). Human MNK1 and MNK2 are approximately 94% identical to their mouse counterparts [68,69]. Shared among all isoforms is a polybasic sequence (PBS) on the N-terminus that confers affinity for eIF4G1/2 and importin α, thus also functioning as a nuclear localization signal (NLS) [44,70,71]. A decrease in the phosphorylation of eIF4E is observed when binding of eIF4G to MNK1 is abrogated [72]. The latter is in keeping with the role of eIF4G as a scaffold protein, bringing MNK1 and eIF4E into close proximity to ensure specific MNK isoforms, wherein a leucine-rich nuclear export sequence (NES) only features in MNK1a, thus enabling CRM1(exportin 1)-mediated nuclear export, and a MAPK-binding domain is exclusive to MNK1a and MNK2a [67,71,73]. A specific amino acid difference in the MAPK-binding motif between MNK1a (LARRR) and MNK2a (LAQRR) contributes, in part, to the preferential binding of p38 MAPK to MNK1a and ERK MAPK to MNK2a. For a comprehensive mapping of residues and motifs within MNK1 and MNK2 that account for preferential MAPK binding, refer to Parra et al. [74,75]. Human MNK1 and MNK2 proteins are phosphorylated on two threonine residues within the T-loop (Thr^209/214^) by ERK1/2 and p38 MAPKs in response to mitogenic- and stress-stimuli, respectively [75]. However, MNK2 shows a higher basal activity than MNK1, which may be explained by features in the C-terminus and the catalytic domain of MNK2 [70,74]. Similarly, MNK2b also displays a high basal activity, although to a lesser extent than MNK2a, whereas MNK1b exhibits a higher basal activity than MNK1a [67,70]. Basal phosphorylation of eIF4E by MNK2a/2b has been suggested to maintain the synthesis of proteins essential for cell survival [67]. 

A unique feature to MNK1/2 is the presence of a DFD (Asp-Phe-Asp) motif, contrary to other kinases in the same superfamily that possess a DFG (Asp-Phe-Gly) motif in the magnesium-binding loop [76]. This results in an unusual DFD-out, auto-inhibited conformation in which the phenylalanine residue flips into the ATP binding pocket hindering the accessibility of ATP [76]. The exclusivity of this domain to MNK1/2 makes it an appealing target for the development of selective inhibitors that stabilize MNK1/2 in its auto-inhibited form and prevent its kinase activity [77].

Expression of particular MNK isoforms may confer different effects on tumor progression. The proto-oncogene SRSF1 (also known as SF2/ASF) regulates *MKNK2* splicing, and is overexpressed in many human tumors including colon, thyroid, small intestine, kidney, and lung [78]. Overexpression of SRSF1 resulted in increased MNK2b and phospho-eIF4E levels, while decreasing MNK2a levels [78]. Importantly, increased levels of MNK2b splice isoform has been reported to mediate resistance to gemcitabine in pancreatic ductal adenocarcinoma [79]. While the MNK2b isoform has been suggested to possess pro-tumorigenic activity, MNK2a is thought to have tumor suppressive effects through phosphorylation, activation, and nuclear translocation of p38, thus causing stress-induced cell death [80]. In contrast, MNK1a and MNK1b have thus far been shown to possess pro-tumorigenic roles [81,82,83].

### 3.3. Differential Cellular Localization of MNK1a/b and MNK2a/b

The presence of an NLS in the N-termini of MNK1/2 and an NES within the C-terminus of MNK1a hints at yet to be uncovered biological functions for MNK1, MNK2, and their splice isoforms. Studies characterizing the splice variants of MNK1/2 have found different subcellular localization of the ”a” and ”b” isoforms [67,70]. MNK1a and MNK2a both predominantly localize to the cytoplasm, despite MNK2a lacking an NES [70]. The latter may be explained by the C-terminus of MNK2a impeding access to its NLS on the N-terminus, consistent with its decreased affinity for eIF4G [70]. On the other hand, MNK1b and MNK2b, which possess a shorter C-termini than the “a” forms, are devoid of an NES and show localization to the nucleus [67,70]. While both MNK1b and MNK2b also phosphorylate eIF4E [67,70], their presence in the nucleus suggests potential divergent functions from the “a” isoforms. MNK2b has been shown to colocalize with nuclear promyelocytic leukemia (PML) protein and eIF4E in the nucleus [70]. PML binds directly to eIF4E through the PML RING domain and reduces the affinity of eIF4E for the m^7^G cap of mRNAs, thus decreasing the nucleocytoplasmic transport of cyclin D1 mRNA and reducing cyclin D1 protein levels [84,85]. These data suggest a role for eIF4E and its phosphorylation by MNK2b in regulating the cytoplasmic export of mRNA.

Interestingly, in nasopharyngeal carcinoma (NPC), astrocytoma, and epithelial ovarian cancer tissues, immunohistochemistry (IHC) was used to show expression of MNK1 or phospho-MNK1 in the nuclei, whereas phospho-eIF4E was more readily observed in the cytoplasm [55,57,61]. In non-small-cell lung carcinoma (NSCLC) patients, high levels of MNK2 were observed in the cytoplasm and correlated with phosphorylated eIF4E levels [86]. Importantly, high levels of MNK2 also correlated with poorer prognosis in NSCLC adenocarcinomas and stage III and IV patients [86]. In patient-derived melanoma samples, increased nuclear and cytoplasmic levels of total and phosphorylated eIF4E has been observed, and high levels of phosphorylated eIF4E were correlated with poor prognosis [58]. Further investigation will be required to determine whether MNK1/2 in the nucleus has yet to be identified substrates, and in turn whether we can better understand the significance of the nuclear localization of MNK1/2 isoforms in tumors.

### 3.4. Identified Substrates of MNK1/2

Despite eIF4E being the only confirmed in vivo substrate of MNK1/2, these two kinases may phosphorylate additional proteins to perform diverse functions. Cytosolic phospholipase A2 (cPLA2) has been suggested to play an important role in tumor angiogenesis and may confer resistance to radiation therapy [87]. Hefner and colleagues reported that MNK1 along with other p38-regulated protein kinases (PRAK1 and MSK1) phosphorylate cPLA2 on serine 727 in vitro and express a dominant negative MNK1 blocked arachidonic acid (an omega-6 fatty acid) release by cPLA2 [88]. Furthermore, the polypyrimidine tract-binding protein (PTB)-associated splicing factor (PSF) has also been shown to be phosphorylated by MNK1 on serines 8 and 283 in vitro [89]. PSF along with p54^nrb^ may play a role in binding AU-rich elements (ARE)-containing mRNAs, such as tumor necrosis factor α (TNFα). This study also demonstrated that MNK1/2 inhibition decreased binding of PSF to TNFα mRNA [89]. PSF is associated with poor prognosis in ER^+^ breast cancer cells, and knockdown of PSF results in a marked reduction of proliferation and increased apoptosis in colon cancer cells [90,91,92]. Another study by Buxade and colleagues identified MNK1 as a potential regulator of tumor necrosis factor α (TNFα) expression through the phosphorylation of hnRNP A1, an ARE-binding protein [93]. hnRNPA1 has been shown to play diverse roles in cancer progression, namely by inducing epithelial-to-mesenchymal transition (EMT; a process by which tumor cells acquire invasive and metastatic properties) in gastric cancer [94,95]. Moreover, two studies have identified Sprouty 2 (Spry2) as an in vitro substrate of MNK1, whereby phosphorylation of Spry2 on serines 112 and 121 stabilizes the protein. Spry2 acts as a negative feedback regulator of RTK signaling by antagonizing growth factors [96,97]. However, Spry2 also possesses pro-tumorigenic effects by triggering increased proliferation in glioblastoma and metastasis in rhabdomyosarcoma [98,99].

## 4. Translational Targets of the MNK1/2-eIF4E Axis

**Figure 3:**
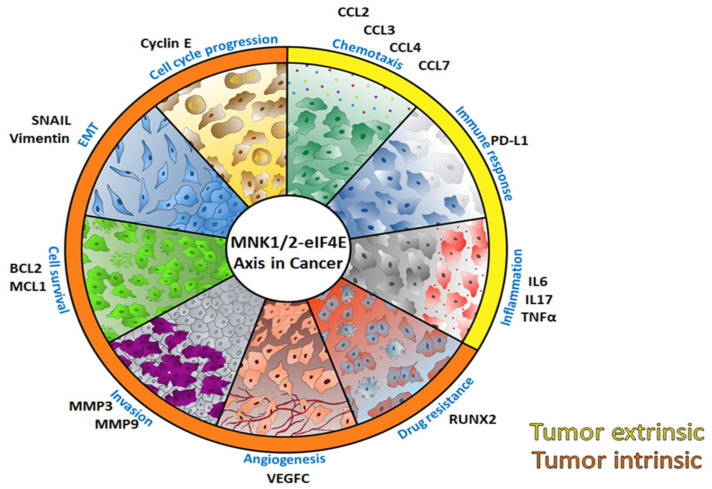
Schematic highlighting the processes that are regulated by the MNK1/2-eIF4E axis. Tumor extrinsic processes are indicated by the yellow outer wheel and tumor intrinsic processes are indicated by the orange outer wheel. Proteins whose mRNA is affected by phosphorylation of eIF4E are in black.

Phosphorylation of eIF4E by MNK1/2 kinases is proposed to enhance the translation of a specific subset of mRNAs that encode proteins with roles in cell survival, invasion, and metastasis [59] (Figure 3). It is important to emphasize that regardless of the controversy surrounding the affinity of phospho-eIF4E for the m^7^G cap [100,101,102,103], an overwhelming body of literature supports that the phosphorylation of eIF4E is essential for its pro-oncogenic and pro-metastatic effects [53,54,58,59,60,62,104]. Furic and colleagues showed that immortalized mouse embryonic fibroblasts (MEFs) harboring a non-phosphorylated eIF4E (eIF4E^S209A/S209A^) are resistant to Ras-induced transformation, and their translation of specific mRNAs is reduced when compared to Wild-Type (WT) MEFs [59]. These include the chemokine *CCL2,* pro-invasive factors matrix metalloproteinases 3 and 9 (*MMP3* and *MMP9*), baculoviral IAP repeat-containing protein 2 (*BIRC2*), and the vascular endothelial growth factor C (*VEGFC*) [59]. The authors also attributed the decreased expression of these proteins to the ability of the mutant mice to be resistant to PTEN loss–induced prostate cancer [59]. Subsequent studies showed that cells lacking phospho-eIF4E were unable to efficiently translate *SNAI1* and *MMP3* and resisted undergoing EMT [105]. Moreover, when MNK1/2 were knocked down in *KIT*-mutant acral melanoma cells, their ability to translate *SNAI1* and *CCNE1* was compromised and their invasive and metastatic properties reduced [62]. Another study highlighted the importance of phospho-eIF4E in promoting the post-translational regulation of *CTNNB1* (encodes β-catenin) in chronic myelogenous leukemia (CML) cells and, thereby, preventing the rise of therapy-resistant stem cells [106]. These studies highlight the contribution of the MNK1/2-eIF4E axis in promoting oncogenicity and invasion. Additional work has led credence to the idea that the phosphorylation of eIF4E also mediates drug resistance. For example, in ER^+^ breast cancer tumors, the phosphorylation of eIF4E on Ser209 promotes tamoxifen-resistance through enhanced translation of RUNX2 [107]. Finally, although much of what we know about the role of the MNK1/2-eIF4E axis has been described in tumor cells, an accumulating body of work suggests an important contribution of this axis in cells of the tumor microenvironment. Pro-metastatic neutrophils isolated from eIF4E^S209A/S209A^-bearing mice also manifest a decreased protein expression of anti-apoptotic factors BCL2 and MCL1, implicating the role of phospho-eIF4E in creating an anti-tumor microenvironment [108].

## 5. Therapeutic Targeting of the Cap-Dependent Translational Machinery

Numerous strategies have been employed to abrogate the oncogenic effects of deregulated mRNA translation. The core proteins of the eIF4F complex are frequently overexpressed in a multitude of cancers including, but not limited to, skin and breast cancer [45,51,109,110]. Currently, inhibiting aberrant mRNA translation in neoplasia involves two treatment modalities: indirectly targeting upstream pathways that converge on the eIF4F complex or directly targeting components of the eIF4F complex. Inhibiting upstream kinases that regulate translation encompasses deregulating the availability of free eIF4E and preventing its phosphorylation-dependent activity. Directly targeting the eIF4F complex aims at decreasing expression of eIF4E, obstructing the interaction between eIF4E and the 5’ cap, disrupting the formation of the eIF4F complex altogether, and inhibiting eIF4A. Emphasis of some of these treatment modalities in melanoma will be reviewed in the subsequent sections.

### 5.1. Directly Targeting the eIF4F Complex

Initial efforts at inhibiting oncogenic translation were geared towards decreasing eIF4E levels and function. Antisense oligonucleotides have been utilized to decrease the intracellular levels of eIF4E [111,112,113,114]. One such antisense anti-cancer drug candidate, LY2275796, has been tested in melanoma, with no evidence of effectiveness [115]. In the phase I clinical trial, LY2275796 was tested in multiple advanced cancers including melanoma, and while administration of LY2275796 at 1000 mg/kg was well-tolerated and was effective at decreasing overall eIF4E levels in tissues, no tumor response was observed [115]. eIF4E levels have also been shown to be regulated by miRNA. Specifically, an increase in miR-768-3p was inversely correlated with eIF4E levels [116]. Overexpression of miR-768-3p was associated with decreased eIF4E levels. The authors showed that the downregulation of miR-768-3p was due to the hyperactivation of the MAPK pathway, a hallmark frequently manifested in melanoma [116]. Targeted inhibitors against BRAF^V600E^ and MEK using PLX4720 and U0126, respectively, decreased the expression of eIF4E through upregulation of miR-768-3p [116]. 

### 5.2. Putting a Cap on eIF4E

While antisense oligonucleotides successfully decrease overall levels of eIF4E, their efficacy in the clinic remains in question. The oncogenic properties of eIF4E, when overexpressed, are dependent on its ability to bind the 5’ cap on mRNAs [117]. Cap analogs have been around for decades and were frequently used in in vitro biochemical assays [118]. However, their use in vivo was initially limited due to issues of cellular permeability and stability [119]. This led to the development of pro-drug versions of cap analogs, which are cell permeable and undergo intracellular processing to active forms [119]. Cap analogs competitively bind to eIF4E and block cap-dependent translation. One such compound, N-7 benzyl guanosine monophosphate tryptamine phosphoramidate pronucleotide (4Ei-1) is a pro-drug that is cell permeable and is converted into the active compound 7-benzyl guanosine monophosphate in cells [119,120]. While clinically untested, multiple groups have demonstrated the anti-neoplastic effects of 4Ei-1 in lung cancer, mesothelioma, and breast cancer [121,122,123]. However, their efficacy in melanoma remains untested.

### 5.3. Disrupting the eIF4E:eIF4G Interaction

As mentioned above, the formation of the eIF4F complex is contingent upon the amount of available or free eIF4E. Activation of the PI3K/Akt/mTOR pathway leads to the hyperphosphorylation of 4E-BPs, resulting in their release of eIF4E, which is in turn free to bind the 5’ mRNA cap and initiate the formation of the eIF4F complex through its interaction with eIF4G [124]. eIF4G, in its role as a scaffold protein, directly binds and stabilizes the association of eIF4E to the 5’cap and recruits eIF4A and the eIF3-40S ribosomal subunit [125]. Hence, impeding the eIF4E:eIF4G interaction is a cogent mechanism to suppress cap-dependent translation. High-throughput screening of 16,000 compounds led to the discovery of the small molecule 4EGI-1, which inhibits the eIF4E:eIF4G interaction and cap-dependent translation [126,127]. Moreover, not only did 4EGI-1 prevent the association of eIF4E to eIF4G, it also enhanced the ability of 4E-BP to sequester eIF4E, thus reducing the amount of eIF4E available for mRNA translation [127,128]. 4EGI-1 demonstrated anti-neoplastic effects on melanoma cells both in vitro and in murine xenografts with a desirable toxicity profile [128,129]. More recently, SBI-756, another small-molecule inhibitor of the eIF4E:eIF4G interaction was identified [130]. Similar to 4EGI-1, SBI-756 promoted the sequestering of eIF4E by 4E-BPs [130]. Upon further characterization, it was demonstrated that SBI-756 impaired eIF4F complex formation in melanoma cells and inhibited the growth of *BRAF, NRAS,* and *NF1*-mutant melanoma cell lines [130]. Furthermore, SBI-756 delayed the onset and decreased the incidence of *NRAS*-mutant melanoma in vivo [130]. Importantly, A375 melanoma cells that acquired resistance to BRAF inhibitors were comparably sensitive to SBI-756 as the parental cell line in vitro [130]. Concomitant administration of PLX4720 and SBI-756 in melanoma xenograft models mitigated the formation of BRAF-inhibitor-resistant tumors [130]. One critical aspect, not covered by the aforementioned study, was to investigate the effects of SBI-756 on invasion and metastasis, as 4EGI-1 can inhibit migration and invasion in vitro [131]. Thus, agents that repress the eIF4E-eIF4G interaction hold the promise of inhibiting high-risk melanomas that are inherently drug resistant, such as *NRAS*-mutant melanomas, or those with acquired resistance to therapy.

### 5.4. Inhibitors of eIF4A

eIF4A is frequently activated in neoplasia, either through mRNA overexpression or suppression of the tumor suppressor protein PDCD4 (programmed cell death 4), which competes with eIF4G for binding to eIF4A [109,132,133,134]. Hence, eIF4A is an attractive target for the development of small molecule inhibitors. Three families of compounds known to inhibit eIF4A include the flavaglines or rocaglamides, pateamine A, and hippuristanol, all of which display potent anti-tumorigenic effects. Flavaglines are natural compounds that are isolated from plants in the genus *Aglaia* and have demonstrated anti-neoplastic effects, with neuro- and cardio-protective properties [135,136,137,138]. Flavaglines exhibit anti-neoplastic effects through inhibition of eIF4A [135]. Crystallographic studies revealed that flavaglines exhibit RNA sequence selectivity, ”gluing” eIF4A onto polypurine sequences in mRNA [136]. Flavaglines have demonstrated inhibitory effects on the CRAF-MEK-ERK signaling pathway via direct inhibition of prohibitins 1 and 2 [137]. The flavagline silvestrol increases the ATPase, helicase, and RNA-binding ability of eIF4A. In doing so, it promotes eIF4A:mRNA interaction in a non-sequence dependent manner and prevents its association with the eIF4F complex [138,139]. In melanoma, silvestrol has been shown to inhibit cell proliferation through increased accumulation of cells in G2/M and promotes autophagy-induced apoptosis [140]. It has been shown to both overcome vemurafenib resistance in *BRAF*-mutant melanoma cells and prevent the rise of therapy-resistant melanoma persister cells [141,142]. Persister cells are a category of cells that are therapy-resistant and may be key in initiating tumor relapse and acquired resistance. Hence, preventing the expansion of persister cells is important in maintaining response to primary therapies. Silvestrol has also been shown to synergize with MEK inhibitors in multiple *NRAS*-mutant melanoma cell lines [143]. Recently, it was also shown that silvestrol exerted its anti-tumor effects by suppressing the expression of the immune checkpoint protein PD-L1, by repressing the translation of *STAT1* mRNA [144]. A current hurdle in the clinical development of silvestrol as an anti-neoplastic agent is resistance mediated by overexpression of the ATP-binding cassette sub-family B1 (*ABCB1*) gene, which codes for the multidrug resistance protein P-glycoprotein (P-gp) [145]. However, researchers are developing compounds that are insensitive to multidrug resistance and demonstrate potent in vivo anti-neoplastic effects [138]. A new compound, FL3, was demonstrated to overcome BRAF-inhibitor resistance in murine models of melanoma [143]. Importantly, flavaglines have started to be tested in clinical trials. The flavagline eFT226 (Zotatifin) has shown efficacy in suppressing the translation of numerous oncogenes including *FGFR1*, *FGFR2*, and *HER2* [146]. Notably, eFT226 significantly suppressed tumor growth in xenograft models harboring amplifications in FGFR1/2 and HER2, and is currently in a phase I/II clinical trial against advanced solid tumor malignancies (NCT04092673) [146,147].

Pateamine A, like silvestrol, increases the ATP hydrolysis, helicase, and RNA-binding activity of eIF4A [148]. However, unlike silvestrol, Pateamine A is not a substrate of P-gp-mediated drug efflux. It irreversibly and covalently binds eIF4A, and is therefore very cytotoxic in vivo [148]. Pateamine A exhibits powerful anti-tumorigenic effects in multiple cancers [149,150]. DMDA-pateamine A, a synthetic derivative of pateamine A, has demonstrated potent inhibition of growth of melanoma xenografts in nude mice with a desirable toxicity profile [150].

In stark contrast to silvestrol and pateamine A, hippuristanol allosterically inhibits eIF4A association with mRNA and its helicase activity in both free form, as well as when eIF4A is incorporated in the eIF4F complex [151]. While the research on hippuristanol in the context of melanoma remains limited, its effects on therapy-resistant melanoma persister cells are comparable to pateamine A and silvestrol [141].

PDCD4 is a tumor suppressor protein that competes with eIF4G and eIF4A and consequently inhibits translation initiation [133,134]. Upon phosphorylation by S6 kinase 1, PDCD4 is tagged by the SCF^βTrCP^ ubiquitin ligase for proteasomal degradation, resulting in the release of eIF4A [133,152,153]. Therefore, increasing PDCD4 expression may provide an avenue for inhibiting oncogenic translation initiation. PDCD4 is negatively regulated by miR-21 [154]. Consequently, antagomirs and the curcumin EF24 analog, which decrease the expression of miR-21, are being investigated for their ability to stabilize the expression of PDCD4. Downregulation of miR-21 in murine melanoma syngeneic grafts by these compounds correlated with increased expression of PDCD4 and led to the formation of smaller lung metastases and increased survival, compared to control mice [154,155,156].

### 5.5. Toggling the Regulation of eIF4E by Targeting Upstream Kinases

Inhibiting eIF4E-mediated mRNA translation by targeting upstream kinases involves either targeting mTOR or MNK1/2 kinases. Inhibitors that interfere with the kinase activity of mTOR aim to prevent the phosphorylation of 4E-BPs and subsequently promote eIF4E:4E-BP complexes, thereby preventing eIF4E from associating with mRNA and the eIF4F complex [157]. While this method is promising, and mTOR inhibitors do exert anti-neoplastic effects, the reality is complicated due to either insufficient inhibition of 4E-BP or through feedback loops [157,158]. mTOR inhibition leads to decreased S6 kinase 1 activity, which results in an increase in pro-survival and proliferative signals through PI3K/Akt signaling [158,159,160,161]. One mode to circumvent the limitations of mTOR inhibitors is to not only prevent eIF4E from associating with eIF4F, but to also prevent the phosphorylation of eIF4E.

As mentioned above, MNK1 and MNK2 are the only kinases able to phosphorylate eIF4E on serine 209, and numerous studies have demonstrated that this phosphorylation event is critical for its oncogenic effects [42,43,44,53,54,57,58,59,60,61,162,163]. Different flavors of MNK1/2 inhibitors are being developed and thoroughly investigated in numerous cancer models. These include ATP-competitive inhibitors and more recently MNK1/2 degraders and allosteric inhibitors of MNK1/2 [77,164]. The most widely tested MNK1/2 inhibitors, CGP57380 and cercosporamide, showed promise in early studies but suffered from significant off-target effects [54,165]. However, more selective MNK1/2 inhibitors are currently under development. In melanoma, MNK1/2 inhibitors were shown to attenuate growth of pulmonary metastases in murine syngeneic grafts [54]. Using CRISPR-Cas9 technology, we recently showed that *BRAF*-mutant melanoma cells devoid of MNK1 were less metastatic in an experimental model of lung metastasis [166]. Comparably, in an experimental model of metastasis, mice treated with SEL201, an ATP-competitive MNK1/2 inhibitor, formed significantly fewer lung metastases than vehicle-control treated mice [166]. Our group has shown that MNK1/2 inhibition using SEL201 blocks the progression of metastatic *KIT* melanoma, concomitant with suppression of eIF4E phosphorylation [62]. We have also shown that use of SEL201 in models of breast cancer is associated with decreased outgrowth of ductal carcinoma in situ and hindered invasive disease progression [63]. Recently, a group reported that SEL201 was also effective in suppressing the growth of acute myeloid leukemia (AML) progenitor cell lines and had no detrimental effects on normal hematopoietic progenitor cells [167]. MNK1/2 inhibitors are being tested as single agents but have also been demonstrated to synergize with other therapeutic agents. Specifically, combined MNK1/2 and MEK inhibition cooperatively killed *NF1*-mutant malignant peripheral nerve sheath tumor (MPNST) cells in vitro and in vivo [168]. In the context of chronic myelogenous leukemia (CML), combined BCR-ABL1 and MNK1/2 inhibition was more effective in suppressing in vivo tumor growth in a xenograft model than was a targeted inhibitor against BCR-ABL1 alone [169]. MNK1/2 inhibition has also shown promise in suppressing the growth and increasing the response to conventional therapy in AML [170,171]. Under the premise that eIF4E-mediated mRNA translation can be modulated by targeting upstream kinases, multiple studies have investigated the combinations of mTOR and MNK1/2 inhibitors. In multiple cancer types, the combined inhibition of mTOR and MNK1/2 enhanced their anti-tumor effects compared to either agent alone [172,173,174,175,176]. In these studies, however, MNK1/2 inhibition was achieved using the CGP57380, and due to its lack of specificity, these results need to be interpreted with caution. However, a recent study demonstrated that selective MNK1/2 inhibition using SEL201 in combination with the mTOR inhibitor rapamycin was synergistic in suppressing the growth of AML progenitor cell lines [167]. This combination treatment represents a promising new avenue, yet to be explored in melanoma.

Importantly, MNK1/2 inhibitors have entered clinical trials for the management of cancers. BAY1143269 is a MNK1/2 inhibitor from Bayer that is currently in phase I clinical trials against advanced or metastatic solid tumors [177,178]. eFT508 (Tomivosertib) is a highly selective MNK1/2 inhibitor in phase I/II clinical trials alone or in combination with the immunotherapeutic agent avelumab [179]. Results from a phase II clinical trial combining eFT508 with PD-L1 inhibitors in microsatellite stable colorectal cancer (MSS CRC) demonstrated robust target engagement with acceptable toxicity, and one patient achieved a partial response lasting almost eight months [179].

The effects of MNK1/2 inhibition are not limited to their anti-neoplastic effects on tumor cells. MNK1/2 inhibition has been shown to elicit desirable, anti-tumor responses in immune cells. MNK1/2 have long been known to be important regulators of soluble factors such as cytokines, chemokines, and growth factors (reviewed in [180]). Such soluble factors have important roles in tumor cells, but also help to shape the tumor microenvironment. In macrophages, the inhibition of MNK1/2 attenuated the production of pro-inflammatory cytokines such as TNF-α, IL-6, and monocyte chemo-attractant protein-1 [181]. Conversely, MNK1/2 inhibition in macrophages stimulated with multiple Toll-like receptor (TLR) agonists also enhanced the production of the anti-inflammatory cytokine IL-10 [181]. Similarly, in TNF-α-stimulated human neutrophils, MNK1/2 inhibition attenuated the secretion of CCL-3, CCL-4, and CXCL8, while the overall mRNA levels of these cytokines remained unchanged [182]. Importantly, the overexpression of MNK1, but not a dominant negative mutant version of MNK1, increased the production of these cytokines [182]. A recent study demonstrated that orthotopic injection of breast tumor cells into phospho-eIF4E deficient transgenic mice led to fewer lung metastases, compared to the same cells injected into wild-type mice [108]. This finding suggested a role for the phosphorylation of eIF4E in cells of the tumor microenvironment. Indeed, the authors showed that the phenotype was due to a decreased expression of the anti-apoptotic proteins BCL2 and MCL1 in pro-metastatic neutrophils [108]. Complementing the results observed in the phospho-eIF4E deficient mice, pharmacological inhibition of MNK1/2 using the less selective inhibitor merestinib, in tumor bearing mice, decreased the levels of neutrophils in the lung [108].

Numerous research groups have investigated the role of MNK1/2 kinases in T-cell development and function. Studies have also demonstrated that inhibition of MNK1/2 kinase activity decreased the production of IL-17 [183]. IL-17 is a pro-inflammatory cytokine produced by a subset of CD4^+^ T helper 17 cells (Th-17). In melanoma, the effects of Th-17 cells and IL-17 production are rather controversial. One study demonstrated significant tumor ablation upon adoptive transfer of tumor specific Th17-polarized cells in vivo, mediated through their production of IL-17 [184]. Another group showed similar results owing to increased recruitment of leukocytes and increased activation of tumor-specific CD8^+^ T cells [185]. Conversely, studies have also shown that Th-17 cells enhance melanoma tumor proliferation and survival through IL-17 production. This resulted in increased production of IL-6 by IL-17 receptor-expressing tumor and tumor-associated stromal cells and concomitantly increased STAT3 signaling and production of pro-survival Bcl-2 and Bcl-xL in melanoma cells [186]. Overexpression of eIF4E, or a constitutively active MNK1, have also been shown to increase the production of RFLAT-1, a transcriptional regulator of CCL5 in T-cells [187]. One study showed that MNK1/2 double knock out transgenic mice had no influence on the development of αβ T cells, T-regs, or natural killer T-cells (NKT) [188]. The study also demonstrated that the CD8^+^ T-cells from mice lacking MNK1/2 did not exhibit deficiencies in response to bacterial and viral infections [188]. In another study, in the context of a T-cell specific PTEN-null lymphoma model, deletion of MNK1/2 resulted in delayed onset of lymphoma, with a complete abolishment of eIF4E phosphorylation [189]. Collectively, these results suggest that inhibition of MNK1/2 promotes favorable anti-tumor conditions intrinsically, while promoting a strong anti-tumor microenvironment, and provides a clear rationale for the relevance and importance of developing MNK1/2 inhibitors. 

## 6. Immunotherapy and Melanoma

Advances in immunotherapy have changed the landscape for the management of melanoma. In BRAF-mutated melanoma, combination immunotherapy achieved similar response rates to those achieved by BRAF and MEK targeted combination therapy [190]. However, in the immunotherapy arm, patients achieved more durable responses over time [190,191,192]. Recent efforts have been put into combining immunotherapy with BRAF and MEK inhibition to further prolong patient overall survival. Multiple preclinical studies have shown immense promise in combining MAPK inhibition with immunotherapy [193,194], and numerous clinical trials are underway in evaluating these combinations (Table 1). A recent phase III clinical trial evaluated the combination of atezolizumab (anti-PD-L1), cobimetinib (anti-MEK), and vemurafenib (anti-BRAF) demonstrated more durable responses and a significant increase in progression-free survival compared to cobimetinib and vemurafenib alone [195]. Recently, efforts have also been put into evaluating the role of the translation machinery in the context of anti-tumor immunity.

One mode by which cancer cells escape immunosurveillance is by modulating the expression of immune suppressive markers. Cells frequently utilize the PD-1/PD-L1 axis to escape the immune system, and thus monoclonal antibodies were developed to block PD-1 or PD-L1 for the treatment of a growing list of malignancies [196,197]. Emerging studies suggest that modulating mRNA translation may be an effective method to increase the efficacy of immunotherapy. One study demonstrated that diminished translation activity decreased the expression of surface PD-L1 in melanoma [144]. Moreover, this effect could be recapitulated in vivo by pharmacologically inhibiting eIF4A [144]. When syngeneic melanoma cells were engrafted into immune competent mice, inhibition of eIF4A using silvestrol resulted in a pronounced delay in tumor growth [144]. The result was attributed to the ability of silvestrol to decrease surface PD-L1 expression and increase tumor infiltration of immune cells [144]. This effect was diminished when the same cells were engrafted into immunocompromised mice, CD8^+^ T-cell-depleted mice, or when PD-L1 was overexpressed in engrafted cells [144]. Similarly, regulating translation through MNK1/2 inhibition has also been credited to a direct decrease in PD-L1 expression. In an aggressive cell model of liver cancer expressing MYC^Tg^;KRAS^G12D^, treatment with eFT508 significantly decreased the translation of and, thereby, surface expression of PD-L1 [198]. This decrease in surface PD-L1 expression was also recapitulated when MYC^Tg^;KRAS^G12D^ were overexpressed in cells harboring non-phosphorylatable eIF4E (eIF4E^S209A/S209A^) [198]. While still in their initial stages, these studies strongly indicate a link between inhibiting translation regulation and increased immune response.

## 7. Conclusions

It is an exciting time to be studying MNK1 and MNK2, as potent pharmacological inhibitors of these kinases are, or have been, in clinical trials (NCT03616834, NCT02605083, NCT03690141, and NCT02439346). While a lot of questions regarding the biology of MNK1/2 remain, the wealth of literature available strongly indicates the pro-tumorigenic role of the MNK1/2-eIF4E axis in numerous cancer types, including melanoma. Further investigation is required to better understand the roles of MNK1/2 with respect to proliferation, metastasis, and the immune system.

## Figures and Tables

**Table 1:** Summary of clinical trials utilizing MAPK pathway targeted therapy in combination with checkpoint immunotherapy.

Immunotherapy Target	Immunotherapy	Targeted Inhibitors	Clinical Trial Identifier	Clinical Phase	Clinical Trial Status
CTLA4	Ipilimumab	Vemurafenib	NCT01400451	Phase I	Terminated
	Ipilimumab	Dabrafenib	NCT02200562	Phase I	Terminated
	Ipilimumab	Dabrafenib; Dabrafenib + trametinib	NCT01767454	Phase I	Completed
	Ipilimumab	BMS-908662	NCT01245556	Phase I	Completed
	Ipilimumab	Vemurafenib	NCT01673854	Phase II	Completed
CTLA4 + PD1	Ipilimumab; Nivolumab; Ipilimumab + nivolumab	Dabrafenib; Trametinib; Dabrafenib + trametinib	NCT01940809	Phase I	Active, not recruiting
	Ipilimumab + nivolumab	Encorafenib + binimetinib	NCT03235245	Phase II	Recruiting
	Ipilimumab + nivolumab	Vemurafenib + cobimetinib	NCT02968303	Phase II	Active, not recruiting
	Ipilimumab + nivolumab	Encorafenib + binimetinib	NCT02631447	Phase II	Active, not recruiting
	Ipilimumab + nivolumab	Dabrafenib + trametinib	NCT02224781	Phase III	Recruiting
PD-1	Nivolumab	Dabrafenib; Trametinib; Dabrafenib + trametinib	NCT02357732	Phase I	Withdrawn
	Nivolumab	Dabrafenib + trametinib	NCT02910700	Phase II	Recruiting
	Pembrolizumab	Vemurafenib + cobimetinib	NCT02818023	Phase I	Active, not recruiting
	Pembrolizumab	Trametinib + dabrafenib	NCT02130466	Phase I/II	Active, not recruiting
	Pembrolizumab	Encorafenib + binimetinib	NCT02902042	Phase I/II	Recruiting
	Pembrolizumab	Dabrafenib + trametinib	NCT02858921	Phase II	Recruiting
	Pembrolizumab	Dabrafenib + trametinib	NCT02625337	Phase II	Unknown/Completed
	Spartalizumab	Dabrafenib + Trametinib	NCT02967692	Phase III	Active, not recruiting
PD-L1	Atezolizumab	Vemurafenib; Vemurafenib + cobimetinib	NCT01656642	Phase I	Active, not recruiting
	Atezolizumab	Cobimetinib	NCT03178851	Phase I	Active, not recruiting
	Durvalumab (MEDI4736)	Dabrafenib; trametinib; Dabrafenib + trametinib	NCT02027961	Phase I/II	Completed
	Atezolizumab	Vemurafenib + cobimetinib; Cobimetinib	NCT03554083	Phase II	Recruiting
	Atezolizumab	Vemurafenib + cobimetinib	NCT02902029	Phase II	Active, not recruiting
	Atezolizumab	Cobimetinib	NCT01988896	Phase I	Completed
	Atezolizumab	Vemurafenib + cobimetinib	NCT02908672	Phase III	Active, not recruiting
